# Oral Morphine Prescribing Practices in Severe Cancer Pain

**DOI:** 10.4103/0973-1075.58458

**Published:** 2009

**Authors:** Barathi B

**Affiliations:** Department of Pain and Palliative Care, St.John's Medical College Hospital, Bangalore, India

**Keywords:** Cancer pain, Oral morphine, Pain relief, Prescribing practices

## Abstract

**Background:**

Nearly one million cancer patients in India need oral morphine for pain relief. Despite doctors prescribing oral morphine in our center, many cancer patients with severe pain found to be not facilitated with adequate pain relief.

**Aim:**

This audit was conducted to look at the “oral morphine prescribing practices for severe cancer pain” at a tertiary care hospital.

**Materials and Methods:**

Twenty case files of patients, who were admitted with severe cancer pain, and receiving oral morphine were analyzed in pre- and posteducational session. Local standards were set to assess the adequacy of pain relief. Deficiency in achieving analgesia was found in preinterventional audit. A clinical audit was conducted before and after the educational session on oral morphine prescribing. The education for doctors and nurses focused on starting patients on morphine, titration, and administering rescue dose. Then local guidelines on oral morphine prescribing were circulated. And analysis of following factors were done following pre- and posteducational session: Pain intensity at the beginning of treatment, starting dose of morphine, increments in morphine dose, number of rescue doses given, and fall in pain intensity at the end of 1 week. The outcomes were compared with the standards.

**Results:**

Preintervention audit showed that only 50% of patients achieved adequate pain relief. Rescue dose was administered in only 20% of patients. While reaudit following the educational session showed that 80% of patients achieved adequate pain relief and 100% received rescue doses.

**Conclusion:**

Educational sessions have significant impact on improving oral morphine prescribing practice among doctors and nurses. It was found failing to administer regular as well as rescue doses resulted in inadequate pain relief in patients receiving oral morphine.

## INTRODUCTION

“Palliative care is a human right!” was the theme for World Palliative Care day celebration for the year 2008, which stressed the importance of people dying without distressing symptoms including pain. Studies found that one million people suffer from cancer pain in India, but only 0.4% of them are able to access oral morphine.[1]

Despite the WHO and other local palliative care services publishing guidelines for cancer pain management, 50% of patients with cancer pain experience inadequate analgesia.[1,3]

Limited availability of strong opioids especially morphine, strict narcotic act,[1,3] lack of awareness among people and healthcare professionals,[1,3,4] and misconceptions and “myths” existing about cancer pain and “morphine” that exist among healthcare professionals[3–5] could be the reasons for inadequate management of cancer pain.

Few strong opioids available in our country are morphine, buprenorphine pethidine, and fentanyl. They are available as oral, sublingual, and injectables, respectively. Suppositories of strong opioids are not available in India; syringe drivers and fentanyl patches are not affordable by everyone in our clinical setting.

Morphine as oral and parenteral preparation is the only option left to treat severe cancer pain in most palliative centers in India. As per WHO recommendation,[6] oral route is the preferred route of administration for cancer pain.

Oral morphine is cost-effective, available as small tablets and liquids so that patients with some dysphagia can swallow it and can be self-administered at home.[4] When compared with parenteral morphine, hypotension and respiratory depression are rare with oral morphine. These factors favor oral morphine as the preferred choice of analgesic for severe cancer pain if patient does not have any problem taking it orally. There is no other suitable alternative to oral morphine in India.[1,4]

More than 90% of our patients in our palliative care center at St. John's Hospital with severe cancer pain receive oral morphine following WHO principles of cancer pain management.[6,7]

Despite doctors prescribing oral morphine in our center, many cancer patients with severe cancer pain do not achieve adequate pain relief. Hence an audit was planned to look at the current standards of practice in oral morphine prescribing patterns at St. John's medical college Hospital, Bangalore. This audit aimed to assess “oral morphine prescribing practices for severe cancer pain.”

## MATERIALS AND METHODS

A clinical audit was conducted with the sample size of 20 in each phase in patients receiving oral morphine for severe cancer pain. Only in-patients were included for the ease of assessing pain relief at a set interval of 1 week from the start of morphine. Numerical scale of 0-10 points had been used to measure pain relief.

A discussion was held with our palliative care team and the following local standards had been set to assess pain relief, which would be appropriate for our clinical setting.

*Standard I*. Eighty percent of patients should achieve adequate pain control at the end of 1 week. Drop of “6” and/or above in 0-10 point numerical scale was determined as adequate pain relief.

*Standard II*. All 100% of patients who has breakthrough pain should receive “breakthrough dose” in addition to regular dose of morphine according to local guidelines [Appendix 1].

**Appendix 1:** 

Departmental guidelines for prescribing oral morphine for severe cancer pain	
Department of Pain and Palliative care	
St. John's Medical College Hospital, Bangalore	
1.	The opioid of first choice for moderate to severe cancer pain is morphine. The optimal route of administration of morphine is by mouth.
2.	The starting dose is 5-10 mg q4 h of immediate release morphine with adequate breakthrough doses.
3.	The breakthrough dose is same as the q4 h dose. The breakthrough dose may be given as often as required.
4.	Patients stabilized on regular oral morphine require continued access to a breakthrough dose to treat “breakthrough” pain.
5.	The total daily dose of morphine should be reviewed daily. The regular dose then can be adjusted to take into account the total amount of breakthrough doses given in a day.
6.	The dose is titrated in this way till patient gets good pain relief. But the aim is to keep the dose optimal and side-effects should not overweigh pain relief.
7.	If pain returns consistently before the next dose is due, the regular dose should be increased (the frequency of dosing should not be increased).
8.	Prescribe anti-emetic p.r.n for vomiting, preferably centrally acting ones like T. Metaclopramide 10-20 mg t.i.d or T. Haloperidol 1.5 mg HS.
9.	Prescribe stimulant laxative alone (T. Bisacodyl 10 mg HS) and/or in combination with a stool softener Syrup cremaffin (milk of magnesia and liquid paraffin) 2 tsp HS regularly.

*Standard III*. In all 100% of patients, the regular increments (titration) of morphine dose should be in line with local guidelines.

After setting the guidelines, analysis of 20 case sheets (32 case sheets was done to get 20 charts that had all required data) of patients with severe cancer pain who were on oral morphine was done by using the data collection sheet [Appendix 2]. The following were assessed: Pain intensity at the beginning of treatment, starting dose of morphine, increments in morphine dose, number of breakthrough doses (BT) given, and fall in pain intensity at the end of 1 week. This result showed that none of the above-mentioned standards had been met and, therefore, achieving pain relief was inadequate.

**Appendix 2:** 

Data collection sheet	
1.	Patient's name, age, sex, hospital no
2.	Diagnosis
3.	Pain intensity at the beginning of treatment (in 0-10 point numerical scale)
4.	Type of pain Nociceptive, neuropathic, mixed
5.	Did the patient start on 5-10 mg q4 h of oral morphine?
6.	Was the breakthrough dose same as fourth hourly dose?
7.	Number of breakthrough doses taken/day
8.	Did the pain intensity reduce within 1 week (in 0-10 point numerical scale)
9.	If not, were increments in line with guidelines?

An educational session on oral morphine prescribing for severe cancer pain was conducted for the doctors and nurses at St. John's hospital and local guidelines were circulated. Only the specialties which treat cancer patients were targeted for educational sessions; they were the department of Medicine, Surgery, Obstetrics and Gynecology, and Orthopedics.

The educational session was one-and-half-hour lecture covering pain assessment, WHO ladder, oral morphine administration, and side-effects of morphine. A brief case presentation of patient who had been receiving oral morphine was made to help the audience to understand the morphine prescription pattern.

Assessing pain with 11-point numerical scale, starting dose of morphine, correct frequency of administration, administering BT doses, titrating morphine according to local guidelines, and common side-effects that can occur had been stressed during the educational session.

There was an additional lecture conducted in those wards within the next 2 weeks following the common lecture with the aim to cover the staffs who could not attend the common lecture.

The local guidelines on oral morphine prescribing was designed, which was adapted from WHO[6,7] and European Association of Palliative Care (EAPC) recommendations,[8] for the management of cancer pain. This was circulated to doctors and nurses of above-mentioned departments and was emphasized during the routine weekly seminars held in these departments.

A period of 2 months was given for adapting the changes in practice. Then reaudit of 20 charts was done and the following were assessed again: Pain intensity at the beginning of treatment, starting dose of morphine, increments in morphine dose, number of BT doses given, and fall in pain intensity at the end of 1 week.

## RESULTS

Twenty charts of patients were analyzed before and after the educational intervention. Male patients (16) were higher compare to female patients (4), and this proportion almost remained the same in reaudit (male 15, female 5).

The age distribution ranged from 26 to 72 years; majority of them were in advanced stage of cancer (18). Six of them were on palliative chemotherapy and four patients were receiving palliative radiotherapy.

### Results of preintervention audit

Assessment of pain at the end of 1 week of treatment had shown that 50% of patients did not achieve adequate pain relief. Adequate pain relief was set as achieving a score of 6 and above in the 0-10 point numerical scale [Table 1].

**Table 1:** Pain relief at the end of one week

Pain score (in numerical scale of 0-10)	Pain relief in % (n = 20)	
	
	Retrospective	Prospective
3-5	50	20
6-8	30	55
9-10	20	25

It was observed that morphine was not given at fourth hourly interval. Often it was given as required, three times a day or four times a day. Only 10% received fourth hourly dose [Table 2]. This might have significantly contributed to inadequate pain relief.

**Table 2:** Frequency of administration

Frequency	(n = 20) in percentage
SOS	35
TID	40
QID	25
Q 4th hourly	10

SOS = Whenever required, TID = Thrice a day, QID = Four times a day

BT dose was not routinely administered. Only 20% had received the BT dose, which is the same as fourth hourly dose of morphine. Of the remaining patients, 20% received injection pethidine 25 mg IV and 10% received injection morphine 2 mg IV as BT dose, which was not compliant with guidelines. And the rest did not receive any BT medicine at all [Table 3].

**Table 3:** Breakthrough dose

Breakthrough dose in mgs	n = 20	
	
	Retrospective (in %)	Prospective (in %)
2.5	10	20
5.0	10	40
10.0	0	30
15.0	0	10

According to guidelines, increments should be made by adding the number of BT dose to the total dose of morphine and adjust the dose as fourth hourly. But it was found in 20% of patients that increments in morphine dose were not according to the guidelines. These preinterventional results revealed that all three set standards had not been met [Table 4].

**Table 4:** Results of preintervention audit

Standards set	Retrospective analysis (%)
I - Adequate pain relief should be achieved in 80% of patients	50
II - Breakthrough doses (BT) should be given in 100% patients	20
III - Morphine increments should be according to local guidelines in 100% of patients	80

### Results of postintervention (Reaudit)

After the educational session, reaudit was done on 20 case files at the end of 2 months. The fall in pain intensity at the end of 1 week has improved from 50% to 80% following the educational session [Table 1].

BT dose was given to all patients, which complied with the guidelines. In all patients, the BT dose was the same as the fourth hourly dose of morphine. The number of BT dose ranged from two to four times a day.

**Graph 1:**
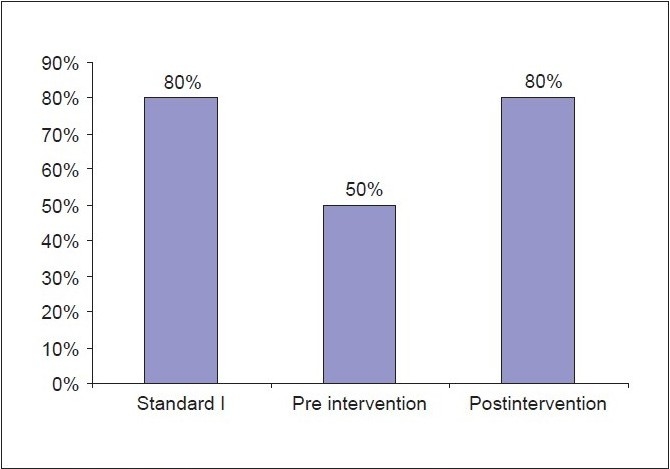
Pain relief comparing pre and post intervention

**Graph 2:**
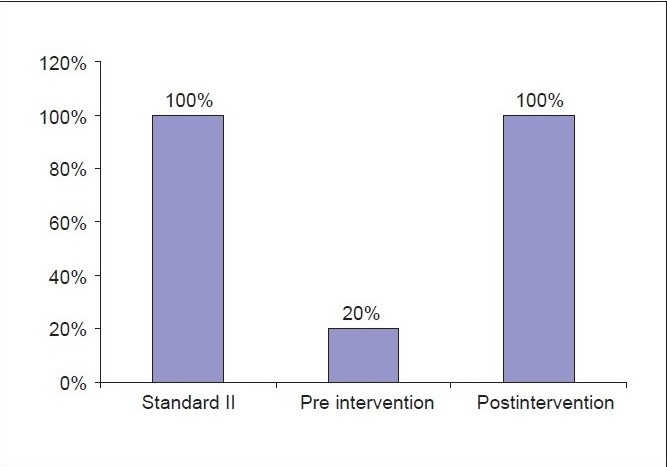
Breakthrough dose administration

**Graph 3:**
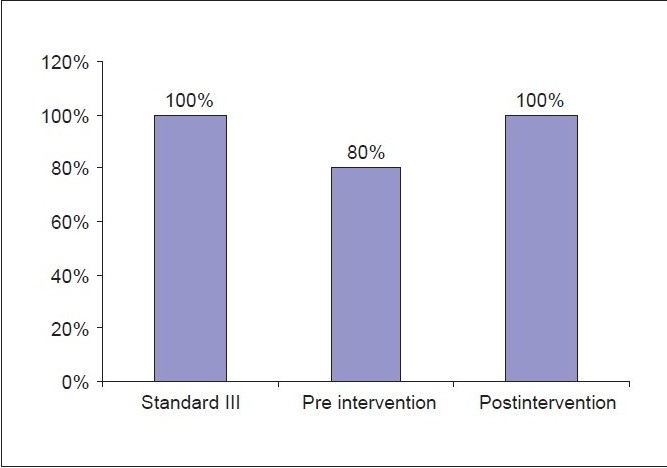
Improvement in morphine increments

In all patients, the increments of morphine dose were in line with local guidelines. The morphine dose was reviewed daily and adjusted according to the number of BT doses given in a day. The audit carried out after the educational session had revealed that all three of the set standards were met [Graphs 1–3].

It was also interesting to find that inappropriate starting dose could have contributed for inadequate pain relief. In 55% cases, the starting dose of morphine was 2.5 mg, which was not recommended by the guidelines.

## DISCUSSION

This audit showed that 80% were able to achieve adequate analgesia; BT doses were administered in 100% patients; and titration of morphine dose was according to local guidelines in 100% patients following educational session.

Inability to achieve adequate pain relief in patients who are taking oral morphine with severe cancer pain is a global problem faced in the world of palliative care.[1] This exists despite local and international guidelines being in place.[2,3]

Zenz *et al*. reported that educational deficit of healthcare professionals and improper allocation of money toward developing knowledge of healthcare professionals are the main barriers of achieving adequate pain relief in cancer.[2]

A similar audit conducted in a hospice at Singapore had revealed that lack of BT doses (two-third) resulted in inadequate pain relief.[9] This had shown similar reasons as in this audit for inadequate pain relief, which are as follows: 14% (35% at St. John's hospital) received only as required doses (PRN) and 52% (10% at St. John's hospital) received only regular doses.

Troller *et al*.[10] who conducted an audit at a specialist cancer hospital showed that lack of BT doses is one of the major drawbacks for inadequate pain relief among cancer in-patients.

Another audit done in New England and South Wales[11] showed that only 43% were receiving BT doses according to the EAPC recommendation, which resulted in inadequate pain relief.

There are no relevant audits conducted in India that is available to compare morphine prescribing practices in India.

The reasons for not administering BT doses were explored during the educational session as that had significant impact on pain relief. These were as follows: Nurses have been too busy and unable to take frequent drug rounds; nurses have thought that they are giving “too much morphine” that could cause serious side-effects like respiratory depression; doctors are hesitant to give BT dose because they thought it can harm kidneys and can cause addiction and respiratory depression.

Few patients had expressed (to nurses) concern that they might develop a “habit” of popping a pill for all their aches and pains. This threw light on an important point that patient education is an essential part for morphine prescription. This may require a separate audit to explore this aspect.

There were a few limitations in conducting this audit. It was difficult to cover all doctors and nurses through educational session for the following reasons: Staffs on long leave, change of duties, and huge staff turnover in our hospital. Ongoing education is necessary to improve the quality of oral morphine prescribing in our hospital. And preinterventional audit had difficulty in retrieving data on pain because of lack of regular pain documentation.

## CONCLUSION

This audit showed that educating healthcare professionals on oral morphine prescribing helped in better prescribing practice, which resulted in achieving adequate analgesia. This audit found that failure to administer morphine at fourth hourly interval and inadequate administration of BT dose in patients receiving oral morphine for cancer pain were the main reasons for inadequate pain relief.
